# Akuter binokularer Visusverlust bei Basilarisaneurysma-bedingter Subarachnoidalblutung

**DOI:** 10.1007/s00347-021-01329-0

**Published:** 2021-02-08

**Authors:** Fabian N. Fries, Philipp Hendrix, Alaa Din Abdin, Berthold Seitz, Chrysovalantis Sourlis, Frederik A. Fries, Ruben Mühl-Benninghaus

**Affiliations:** 1 Klinik für Augenheilkunde, Universitätsklinikum des Saarlandes UKS, Kirrberger Str. 100, Gebäude 22, 66424 Homburg/Saar, Deutschland; 2 Klinik für Neurochirurgie, Universitätsklinikum des Saarlandes UKS, Homburg/Saar, Deutschland; 3 Klinik für Neuroradiologie, Universitätsklinikum des Saarlandes UKS, Homburg/Saar, Deutschland

## Anamnese

Ein 56-jähriger Mann stellte sich in der Notaufnahme eines peripheren Krankenhauses mit akut aufgetretenen Vernichtungskopfschmerzen vor. Vor Ort konnte die Diagnose einer Subarachnoidalblutung (SAB) ohne motorische oder sensible Ausfälle gestellt werden. Zur weiteren Therapie wurde der Patient analgosediert und intubiert mit dem Rettungshubschrauber an das Universitätsklinikum des Saarlandes als nächstgelegenes Haus der Maximalversorgung verlegt. Dort konnte initial ein Coiling eines rupturierten Aneurysmas der A. basilaris durchgeführt werden. Unmittelbar nach Erwachen aus der Narkose gab der Patient ein binokulares Verschwommensehen an. Ein Augenarzt wurde konsiliarisch hinzugezogen, um eine Beurteilung am Patientenbett durchzuführen. Der Patient befand sich zu dieser Zeit in stationärer Behandlung auf der neurochirurgischen Intensivstation.

## Klinischer Befund

Eine erste orientierende Untersuchung des liegenden Patienten mit der Handspaltlampe zeigte klare Hornhautverhältnisse, eine tiefe und reizfreie Vorderkammer sowie eine altersentsprechend klare Linse. Der Augeninnendruck wurde palpatorisch rechts wie links auf 15 mm Hg geschätzt. Die Sehschärfe betrug beidseits Handbewegung (HBW). Subjektiv schilderte der Patient, alles sehr verschwommen zu sehen. Ophthalmologische Vorerkrankungen seien nicht bekannt, letzte Woche sei er noch mit dem Auto zur Arbeit gefahren. Der Einblick schien aufgrund ausgeprägter Miosis unter mit Perfusor applizierter Opioidtherapie an beiden Augen deutlich reduziert. Außer einem flüchtigen Rotschimmer konnten keine retinalen Strukturen dargestellt werden.

## Wie lautet ihre Diagnose?

**Diagnose:** Binokulare *Glaskörperblutung bei* rupturiertem Basilarisaneurysma mit *Subarachnoidalblutung* (*Terson-Syndrom*)

**Figure 1:**
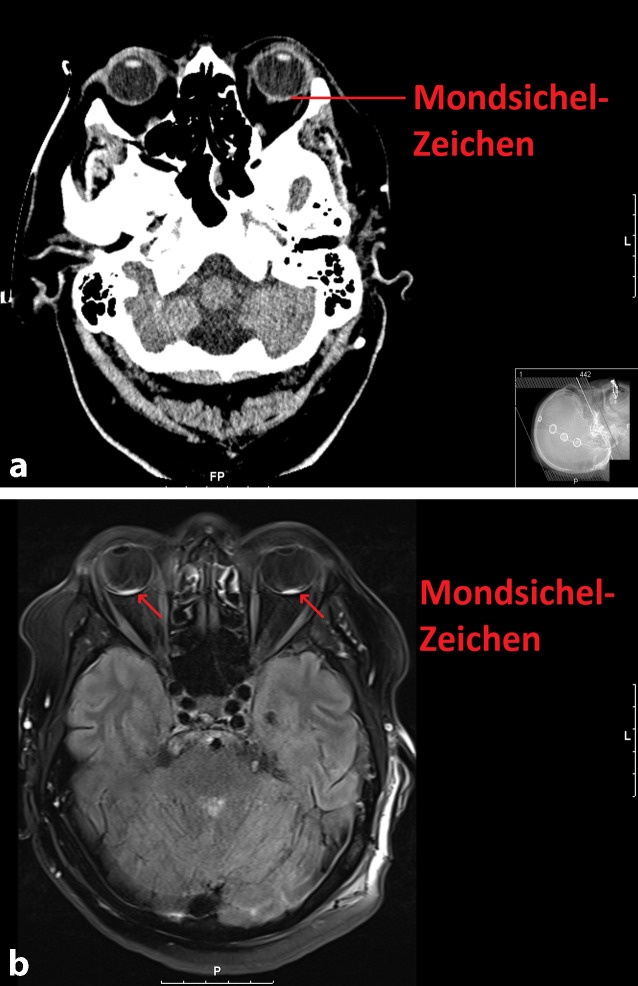

**Figure 2:**
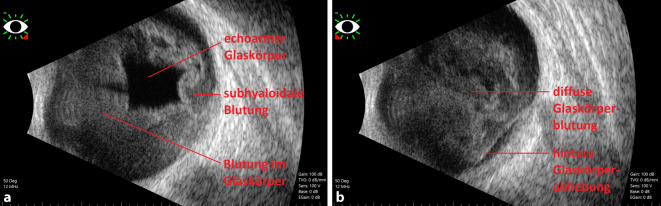

Initial entschloss man sich, aufgrund des unmittelbar nach der Intervention aufgetretenen binokularen Visusverlustes rasch eine weitere Bildgebung durchzuführen um eine erneute (Aneurysma‑)Blutung oder eine neu aufgetretene Ischämie auszuschließen. Der vorläufige Magnetresonanztomographie(MRT)-Befund beschrieb *frische Ischämien in der linken Inselregion und rechten Hemisphäre des Kleinhirns, kein Anhalt für Restperfusion des gecoilten Basilarisaneurysmas, schmales Subduralhämatom bihemisphärisch (parietal rechts betont), älterer Parenchymdefekt in den Stammganglien rechts* sowie eine *superfizielle Siderose infratentoriell*, ohne hinreichende Erklärung der klinischen Symptome in Form des binokularen Visusverlustes (Abb. 1b). Nach mehrfacher lokaler Applikation von Mydriatikum und fehlendem Funduseinblick wurde mithilfe einer mobilen Untersuchungseinheit eine standardisierte ophthalmologische Echographie angeschlossen. Es konnte die Diagnose einer binokularen Glaskörperblutung gestellt werden (Abb. 2a, b). Das sog. Mondsichel-Zeichen ließ sich in der retrospektiven Bildbetrachtung unter Kenntnis der Glaskörperblutung in der kranialen Computertomographie (CCT) und MRT als schmale Signalalteration am Augenhintergrund abgrenzen (Abb. 1b).

## Therapie und Verlauf

Wir leiteten eine lokale Therapie mit 1%iger Prednisolonacetat Augentropfensuspension 8‑mal/Tag ein. Da der Allgemeinzustand des Patienten sich in den folgenden Tagen deutlich besserte, konnte er zur neurologischen Rehabilitationstherapie in eine heimatnahe Klinik verlegt und an die dortige universitäre ophthalmologische Nachsorge angebunden werden. Nach 4 Wochen wurden dort beide Augen bei unzureichender Resorption der Glaskörperblutung konsekutiv vitrektomiert. Zum Zeitpunkt der letzten Nachsorgeuntersuchung, 9 Monate nach dem Blutungsereignis, besteht am rechten Auge eine Sehschärfe von 1,0 und am linken Auge eine Sehschärfe von 0,8.

## Diskussion

Aus pathophysiologischer Sicht werden beim Terson-Syndrom verschiedene Mechanismen diskutiert. Ein älterer Erklärungsversuch vermutete eine direkte Ausbreitung der intrakraniellen Blutung in der Nervenfaserscheide entlang des von Liquor umgebenen N. opticus bis in den Bulbus. Heute geht man davon aus, dass die intrakranielle Blutung zu einem Druckanstieg und einer Dilatation der Optikusscheiden führt. Die hierdurch bedingte Kompression der V. centralis retinae induziert eine akute orbitale und uveale Venostase, was schließlich zum Platzen von kleineren retinalen Gefäßen führt und als Ursache der intraokularen Blutung gesehen wird. Letzteres Modell wurde jüngst unter anderem durch intraoperative Beobachtungen im Rahmen einer Fallserie von 10 Patienten mit Terson-Syndrom bestärkt [1, 4].

Kang et al. beschreiben ein Kollektiv von 31 Patienten mit Aneurysma-bedingter SAB ohne ophthalmologische Symptome, die einer routinemäßigen strukturierten augenärztlichen Untersuchung zugeführt wurden. In ihrer retrospektiven Studie zeigte sich ein signifikanter Zusammenhang zwischen dem gehäuften Auftreten von retinalen Blutungen und dem initialen klinischen Schweregrad der Subarachnoidalblutung, klassifiziert nach Hunt und Hess (Grad 1 bis 5, geringerer Grad mit besserer Prognose). Unter den Patienten mit einem Schweregrad von 3 lag bei 16,7 % eine intraokulare Blutung vor, welche zur Diagnose eines Terson-Syndroms führte. Bei einem Schweregrad von 4 stieg die Prävalenz der intraokularen Blutungen bereits auf 58,3 % an. Sie empfehlen aus diesem Grund, insbesondere Patienten mit prognostisch ungünstiger Ausgangslage (entspricht höherem Schweregrad nach Hunt und Hess) einer augenärztlichen Untersuchung zuzuführen, auch wenn ophthalmologische Beschwerdefreiheit besteht, um eine potenziell notwendige ophthalmologische Therapie nicht zu verzögern [5].

Einen interessanten Aspekt bietet auch die bei SAB routinemäßig durchgeführte Computertomographie (CT). Erfahrene Diagnostiker können durch Beurteilung des „Mondsichel-Zeichens“ (engl. „crescent-sign“) auf das Vorhandensein einer retinalen Blutung schließen (Abb. 1a, b). In einer Studie der Mayo Clinic Florida, welche sich auf retinale Blutungen mit einem Durchmesser von 2 mm oder mehr fokussiert, wurden eine Sensitivität von 85,7 % und eine Spezifität von 99,1 % erreicht. Die Autoren sehen hierin eine effiziente Selektionsmöglichkeit und Alternative zur initialen ophthalmologischen Untersuchung am Patientenbett als klinische Routine [6].

Es existieren zwar vereinzelte Fallberichte über eine Lasermembranotomie bei bilateralem Terson-Syndrom, die Indikationsstellung scheint aber bei isolierter subhyaloidaler Blutung aussichtsreicher. Eine verzögerte Resorption der intraokularen Blutung kann zur Bildung von epiretinalen Membranen, Schäden der Retina durch Blut- oder Eisenablagerungen (Siderose) aus Abbauprodukten des Blutes sowie zu einer proliferativen Vitreoretinopathie führen [2, 3]. Therapie der Wahl bei unzureichender Resorption ist die Pars-plana-Vitrektomie. Insbesondere bei beidseitigem Terson-Syndrom sollte mit der Operation nicht zu lange zugewartet werden. Es wird empfohlen, wenigstens 1 Auge bereits nach 1 bis 3 Monaten zu vitrektomieren, um gleichzeitig auch die Rehabilitation der ansonsten visuell stark eingeschränkten Patienten zu fördern, sofern dies unter Berücksichtigung des Allgemeinzustandes möglich ist [1].

## Fazit für die Praxis

- Obgleich einem akuten binokularen Visusverlust meist eine neurologische Genese zugrunde liegt, sollte differenzialdiagnostisch auch an die binokulare Glaskörperblutung im Rahmen eines Terson-Syndroms gedacht werden.
- Das „Mondsichel-Zeichen“ (engl. „crescent-sign“) in der Computertomographie bzw. Magnetresonanztomographie kann erste Hinweise auf das Vorliegen von retinalen Blutungen liefern.
- Eine standardisierte ophthalmologische Echographie ist bei erschwertem Funduseinblick und fehlender neurologischer Ursache wegweisend.
- Bei unzureichender spontaner Resorption der Blutung sollte zeitnah eine Vitrektomie zur Visusrehabilitation durchgeführt werden.
